# Little ones can do big things: Small molecule inhibitors target PTPN2/PTPN1 for tumor immunotherapy

**DOI:** 10.1002/mco2.567

**Published:** 2024-05-29

**Authors:** Junyu Wang, Shugang Qin, Anren Zhang

**Affiliations:** ^1^ Department of Rehabilitation Medicine Shanghai Fourth People's Hospital, School of Medicine, Tongji University Shanghai China; ^2^ Department of Critical Care Medicine, Frontiers Science Center for Disease‐related Molecular Network State Key Laboratory of Biotherapy and Cancer Center, West China Hospital, Sichuan University Chengdu China

## Abstract

AC484 was developed by designing compounds based on the PTPN2 protein structure. AC484 enhances antitumor immunity through multiple mechanisms: increasing tumor sensitivity to IFN‐γ, improving T‐cell functions, stimulating tumor microenvironment inflammation, expanding TCR diversity, and preventing T‐cell exhaustion. Interestingly, the efficacy of AC484 was also mediated by CD8+ and NK cells.

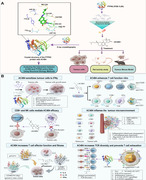

1

A recent study published in *Nature* by Baumgartner et al.^1^ reported a novel tumor immunotherapy strategy by targeting inhibition of the active sites of protein tyrosine phosphatases nonreceptor type 2 (PTPN2) and protein tyrosine phosphatases nonreceptor type 1 (PTPN1) by a small molecule inhibitor ABBV‐CLS‐484 (AC484), demonstrating strong potential for triggering powerful antitumor immunity. Beginning with a structure‐based compound design, the researchers conducted a screening and successfully identified small molecule inhibitor AC484, which primarily works by employing the following antitumor mechanisms: (1) AC484 sensitized tumor cells to IFN‐γ; (2) AC484 promoted the activation of human immune cells and enhanced T‐cell function; (3) CD8^+^ and NK cells were involved in mediating the antitumor effects of AC484; (4) AC484 induced proinflammatory remodeling of the tumor microenvironment, playing a role in antitumor immunity; (5) AC484 increased TCR diversity in tumors; (6) AC484 led to the generation of specific CD8^+^ effector T cells; (7) AC484 prevented T‐cell exhaustion; (8) AC484 enhanced T‐cell effector functions and adaptability. Looking ahead, the investigation of small molecule inhibitors targeting PTPN2/PTPN1 lays a crucial theoretical and experimental foundation for developing novel immunotherapeutic.

Within the protein tyrosine phosphatase family, PTPN2 (TC‐PTP) and PTPN1 (PTP‐1B) function as critical negative regulators in cytokine signaling and TCR signaling pathways, thereby playing a significant role in controlling inflammation.^2^ Research indicates that eliminating PTPN2 from cancerous cells improves the outcomes of immunotherapy by increasing the sensitivity of the tumor to IFN‐γ.^3^ However, developing small molecule inhibitors targeting PTPN2/N1 is challenging due to the highly polar active site of the phosphatase, often deemed as a difficult drug target. Baumgartner et al.^1^ sought to overcome this by employing protein structure‐based compound design. Their initial compound, A‐650, showed potent biochemical inhibitory effects on both PTPN1 and PTPN2, but had poor physicochemical properties for drug development. Further optimization led to the discovery of AC484. Structural analysis through advanced biology methods has clarified the interaction between AC484 and the PTPN1/PTPN2 complex, highlighting crucial bonding at the Cys216 site that leads to substantial biochemical efficacy in the low nanomolar range for both enzymes. The potency of AC484 in relation to PTPN1 and PTPN2 is evidenced by its low nanomolar IC50 values at 1.8 nM for PTPN2 and 2.5 nM for PTPN1, thereby increasing its cellular effectiveness. As an amphoteric compound, AC484 displayed characteristics of low plasma protein binding and low clearance rates. It was primarily excreted through the kidneys and bile without showing hepatic clearance. In mouse models, AC484 demonstrated dose‐dependent linear increases and good target coverage. Consequently, administering AC484 orally is capable of attaining therapeutically effective concentrations.

Additionally, AC484 triggers the activation of primary mouse T cells in laboratory conditions, elevating the occurrence of CD69^+^ and CD25^+^ T cells, alongside boosting the secretion of proinflammatory cytokines such as IFN‐γ and tumor necrosis factor‐alpha.^3^ The concurrent blockade of PTPN2 and PTPN1 by AC484 facilitates a more potent activation of T cells than the inhibition of either phosphatase alone. Experiments involving preactivated human whole blood exposed to AC484 have demonstrated an uptick in IFN‐γ‐stimulated STAT1 phosphorylation, an increase in CXCL10 secretion, and an enhancement in T‐cell activation and operational capacity. Baumgartner et al.^1^ observed significant changes in immune infiltration and cell states after AC484 treatment, with mice undergoing treatment showing deeper immune cell penetration into tumors. Single‐cell transcriptomic analysis revealed that AC484 induced significant changes in the proportion and transcriptional states of various immune subgroups, promoting inflammatory remodeling of the tumor microenvironment and enhancing antitumor immunity. Researchers also found enhanced antigen presentation and increased TCR diversity in tumor cells treated with AC484. Interestingly, AC484 treatment fostered a unique state of CD8^+^ effector T cells characterized by high cytotoxicity and effector gene expression. Importantly, the facilitation of JAK–STAT signaling by AC484 results in both transcriptional and epigenetic modifications within effector CD8^+^ T cells, improving their functional capacity while reducing the incidence of T cell exhaustion. Additionally, AC484 markedly augments the mitochondrial mass in T cells, leading to significant alterations in both epigenetic profiles and metabolic activity. Furthermore, AC484 administration resulted in a dose‐dependent increase in GZMB expression in NK cells, significantly enhancing their ability to kill YAC‐1 cells.

AC484 stimulates immune cells, markedly enhancing their capacity to eliminate cancer cells, and exhibits potent anticancer activity in a mouse model that shows resistance to programmed cell death protein 1 (PD‐1) inhibitors. AC484 is presently being assessed in clinical trials involving individuals with advanced solid malignancies. Immunotherapy using immune checkpoint blockade is a strategy that leverages the human immune system to treat malignant tumors. Immune checkpoint inhibitors (ICIs), represented by inhibitors of PD‐1 and its ligand PD‐L1, have achieved breakthrough progress in tumor immunotherapy, particularly in the treatment of lung cancer, renal cancer, and melanoma.^4^ Interestingly, AC484 has been observed to trigger both the infiltration and activation of human immune cells, promoting anticancer activity through the amplification of crucial immune signaling cascades. This action mirrors the effects seen in T cells subjected to treatment with anti‐PD‐L1 inhibitors and interleukin‐2. However, ICIs, especially PD‐1/PD‐L1 inhibitors, face clinical challenges such as low response rates and resistance in tumor patients, with immunosuppressive cancer microenvironments identified as a major obstacle.^5^ Relative to anti‐PD‐1 inhibitors, AC484 demonstrated distinct benefits by promoting a rise in CD45^+^ immune cell infiltration, a comparative reduction in regulatory T cells, an augmentation of infiltrating NK cells, and an elevated proportion of NK cells and CD8^+^ T cells that express GZMB. Additionally, AC484 increased TCR diversity within tumors; it led to a proinflammatory tumor microenvironment supportive of antitumor immunity, which is inconsistent with the tumor microenvironment induced by anti‐PD‐1 inhibitors. In contrast to the monotherapy with PD‐1 blockade agents, the synergistic application of AC484 alongside anti‐PD‐1 inhibitors may significantly bolster T cell‐mediated anticancer immunity, potentially leading to superior therapeutic results.

In summary, this study unveils a novel antitumor immunotherapy strategy. Employing structure‐based design, the team synthesized the small molecule inhibitor AC484, specifically targeting PTPN2/PTPN1. The efficacy of AC484 against cancer through immune modulation was subsequently confirmed via numerous biochemical tests. The application of AC484 is significantly effective in a range of preclinical study, even in models unresponsive to PD‐1 inhibitor therapy. AC484 triggers immunological responses through a variety of pathways. Currently, AC484 is being evaluated in Phase I clinical trials, administered both as an independent treatment and in conjunction with anti‐PD‐1 therapies for solid tumor management. This paper offers pivotal insights for ongoing research in tumor immunotherapy and the development of novel antitumor small molecules. AC484, a small molecule inhibitor, was derived through continuous optimization based on the protein structures of PTPN2/PTPN1. Compared with traditional drug development, strategies for drug design and optimization based on protein structures have been shown to enhance the efficiency of drug research and development. Future studies, taking cues from the methods employed in this research, on the design and optimization of small molecule inhibitors targeting certain tumor‐related protein targets will further aid in the development of innovative cancer drugs. Simultaneously, future research should focus on refining AC484's drug design, dosage, and combination therapy strategies to enhance its antitumor efficacy. This could involve pharmacochemical modifications or pairing with other immunotherapeutic agents, such as ICIs, CAR‐T cell therapy, and mRNA vaccines.

## AUTHOR CONTRIBUTIONS

J. Y. W. and S. G. Q. wrote the manuscript and made the figure. A. R. Z. conducted the supervision and revised the manuscript. All authors have read and approved the article.

## CONFLICT OF INTEREST STATEMENT

The authors declare that they have no conflict of interest.

## FUNDING INFORMATION

This study was supported by the National Natural Science Foundation of China (No. 82300113), China Postdoctoral Science Foundation (No.2022M722269), and Tongji University Affiliated Shanghai Fourth People's Hospital Research Launch Special Fund (No. sykyqd0201).

## ETHICS STATEMENT

Not applicable.

## Data Availability

Not applicable.
